# Supplementary material for: The causal role of circulating inflammatory markers in osteoporosis: a bidirectional Mendelian randomized study
Source: Front Immunol. 2024 Jul 18;15:1412298. doi: 10.3389/fimmu.2024.1412298 (PMC11291241; doi:10.3389/fimmu.2024.1412298)
Supplement: Supplementary file 1 [file DataSheet_1.zip › supplementary material/OSTEOPOROSIS-CIM/GCST90274768.tsv.gz_forest.pdf]

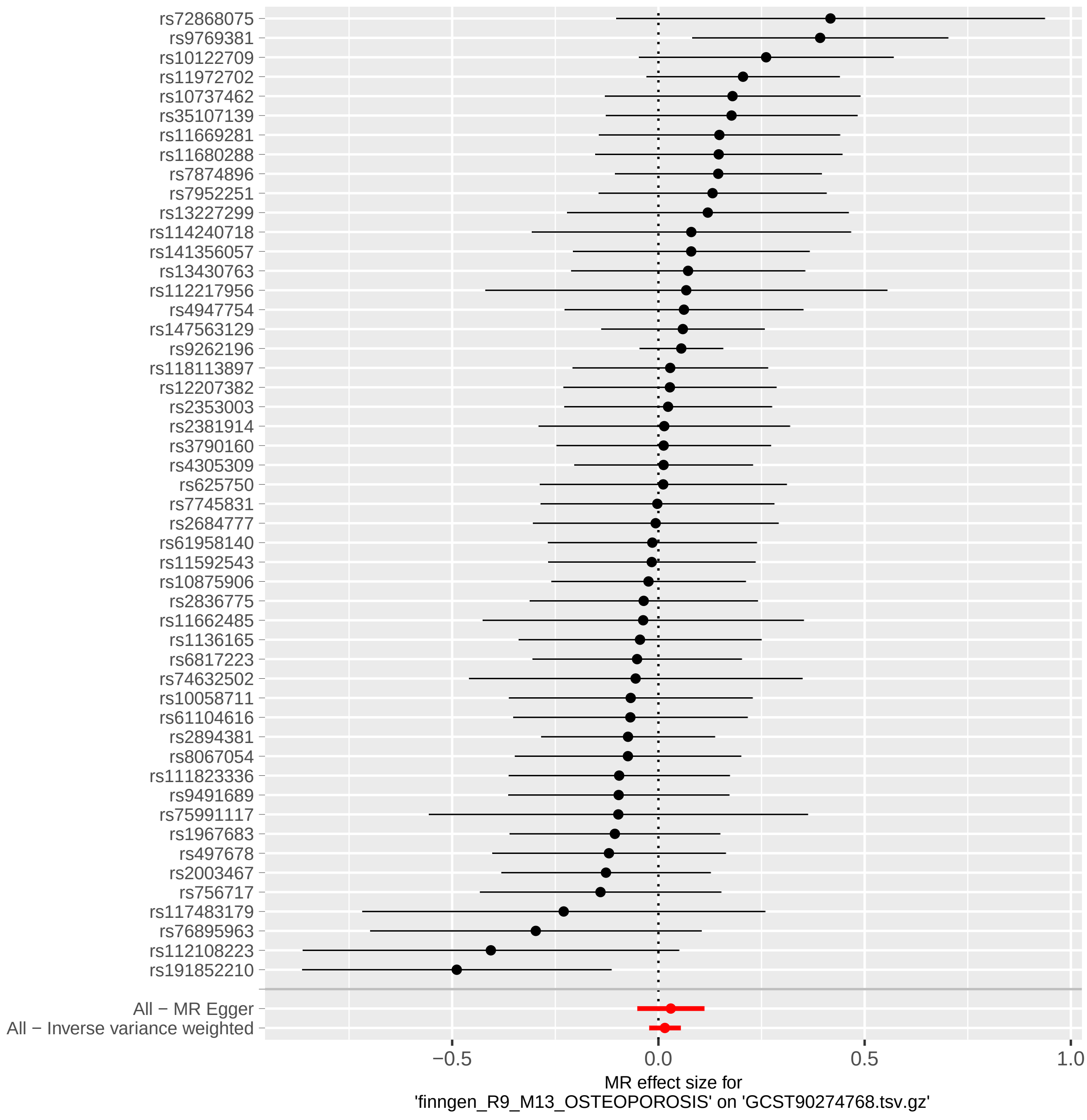
rs72868075
rs9769381
rs10122709
rs11972702
rs10737462
rs35107139
rs11669281
rs11680288
rs7874896
rs7952251
rs13227299
rs114240718
rs141356057
rs13430763
rs112217956
rs4947754
rs147563129
rs9262196
rs118113897
rs12207382
rs2353003
rs2381914
rs3790160
rs4305309
rs625750
rs7745831
rs2684777
rs61958140
rs11592543
rs10875906
rs2836775
rs11662485
rs1136165
rs6817223
rs74632502
rs10058711
rs61104616
rs2894381
rs8067054
rs111823336
rs9491689
rs75991117
rs1967683
rs497678
rs2003467
rs756717
rs117483179
rs76895963
rs112108223
rs191852210
All − MR Egger
All − Inverse variance weighted
−0.5
0.0
0.5
1.0
MR effect size for
'finngen_R9_M13_OSTEOPOROSIS' on 'GCST90274768.tsv.gz'
